# Optimization of Collagen Extraction from Fish Scales Using Tris-Glycine Buffer: A Taguchi Methodological Approach

**DOI:** 10.3390/md22120562

**Published:** 2024-12-17

**Authors:** Mokgadi Ursula Makgobole, Stanley Chibuzor Onwubu, Abayomi Baruwa, Nomakhosi Mpofana, Zodidi Obiechefu, Deneshree Naidoo, Andile Khathi, Blessing Mkhwanazi

**Affiliations:** 1 School of Laboratory Medicine and Medical Sciences, College of Health Sciences, University of KwaZulu-Natal, Durban 4001, South Africa; khathia@ukzn.ac.za; 2 Department of Somatology, Faculty of Health Sciences, Durban University of Technology, Durban 4001, South Africa; nomakhosim@dut.ac.za; 3 Department of Chemistry, Faculty of Applied Sciences, Durban University of Technology, Durban 4001, South Africa; profstan4christ@yahoo.com (S.C.O.); zodidi68@gmail.com (Z.O.); deneshree.naidoo3@gmail.com (D.N.); 4 Department of Biotechnology and Food Science, Durban University of Technology, Durban 4001, South Africa; abayomi.baruwa@gmail.com; 5 Discipline of Dietetics and Nutrition, College of Agriculture, University of KwaZulu-Natal, Durban 3201, South Africa; mkhwanazib@ukzn.ac.za

**Keywords:** collagen extraction, fish scales, Tris-Glycine buffer, optimization, Taguchi method, sustainable biomaterials, yield

## Abstract

Collagen, a critical biomaterial with wide applications in pharmaceuticals, cosmetics, and medical industries, can be sourced sustainably from fish scales. This study optimizes the extraction of collagen using Tris-Glycine buffer from fish scales via the Taguchi method. Various extraction parameters—buffer concentration, temperature, pH, and time—were evaluated to identify optimal conditions. Under optimal conditions (0.5 M of acetic acids, volume of acids of 100 mL, soaking time of 120 min, and Tris-Glycine buffer of 10 mL), the results demonstrate that temperature and buffer concentration significantly influence collagen yield, with a collagen purity of 17.14 ± 0.05 mg/g. *R*^2^ value of 73.84% was obtained for the mathematical model). FTIR analysis confirmed the presence of characteristic collagen peaks at 1611 cm^−1^ (amide I), 1523 cm^−1^ (amide II), and 1300 cm^−1^ (amide III), indicating the successful extraction of type I collagen. SDS-PAGE analysis revealed a protein banding pattern consistent with the molecular weight of collagen, and amino acid analysis shows high percentages of glycine (20.98%), proline (15.43%), and hydroxyproline (11.51%), implying fibrous collagen structures. The finding suggests that the Taguchi method offers an efficient and sustainable approach for collagen extraction, reducing waste from fish processing industries. Nevertheless, there is a need for further experimental validation to align with mathematical modeling on the optimized conditions.

## 1. Introduction

Collagen is the most abundant structural protein in animals, constituting approximately 30% of the total protein in mammalian bodies [1]. It plays an essential role in maintaining the structural integrity of tissues, providing strength and flexibility to bones, tendons, skin, cartilage, and ligaments [2]. According to San Antonio, Jacenko [3], collagen is essential for hemostasis, wound healing, angiogenesis, and biomineralization. Conversely, when collagen is dysfunctional, it can lead to conditions such as fibrosis, atherosclerosis, cancer metastasis, and brittle bone disease [3]. Due to its remarkable biocompatibility and bioactivity, collagen has gained significant interest for its use in various industries, particularly in the pharmaceutical, cosmetic, and biomedical fields [4]. Applications range from wound healing [5,6], tissue engineering [7,8], bio fillers [9,10] to cosmetic products [11] and nutraceuticals [12]. In addition, collagen possesses important characteristics, including high tensile strength [13], low antigenicity [14], and excellent biocompatibility [15]. Collagen promotes blood platelet coagulation, influences cell differentiation, and plays a role in wound healing [16].

Traditionally, collagen has been sourced from terrestrial animals such as cows, pigs, and sheep [17]. However, concerns regarding zoonotic disease transmission, religious restrictions, and growing awareness about sustainability and environmental impact have prompted the search for alternative collagen sources [18]. From a health perspective, collagen source from marine such as fish scales seems not to have disease transmission [19,20]. Fish scales, an underutilized byproduct of the seafood industry, offer a sustainable and eco-friendly source of collagen [21]. Fish scale-derived collagen, particularly from species like tilapia, has been found to have comparable physicochemical properties to mammalian collagen. Utilizing fish scales not only reduces waste from fish processing but also aligns with the global push toward sustainable development and the circular economy [9]. Moreover, the by-products are low cost and the need to minimize fish industry waste’s environmental impact paved the way for the use of discards in the development of collagen-based products with remarkable added value [20].

While fish scales offer a promising source of collagen, the extraction process poses significant challenges. For instance, collagen in fish scales is embedded within a dense matrix of calcium salts [22], making efficient extraction difficult. Traditional methods often involve the use of strong acids or enzymes, which can degrade the collagen structure or result in low yields. Moreover, these methods can be time-consuming and expensive, limiting the industrial scalability of fish-scale collagen extraction [23,24]. The use of Tris-Glycine buffer for collagen extraction offers an innovative alternative. Tris-Glycine is a widely used buffer in biochemical research, known for its ability to maintain a stable pH over a wide range of conditions [25], which is crucial for preventing collagen degradation during extraction. By using Tris-Glycine, the extraction process can be more controlled, reducing the need for harsh chemicals and improving the purity and yield of the collagen obtained.

Although a vast number of studies are available on the extraction of collagen from marine sources, the optimization and the study of the effect of Tris-Glycine on the extraction of collagen from the skin of the tilapia fish is lacking. Additionally, optimization of the extraction process is essential to maximize collagen yield and maintain its bioactivity [26]. The Taguchi method, a statistical approach used for optimizing complex processes, is well-suited for this purpose. By systematically varying key factors such as buffer concentration, temperature, pH, and extraction time, the Taguchi method allows the identification of the optimal conditions with a minimal number of experiments [27]. This approach reduces time and resource consumption while ensuring high-quality results [28]. Several studies have successfully used the Taguchi method to optimize biochemical processes. For instance, Mokrejš, Gál [29] optimize the processing of the MDCM by-product into gelatins using Taguchi method, achieving enhanced yield and quality of the gelatins. Taguchi design and response surface methodology (RSM) was also applied to optimize the extraction parameters, such as acetic acid concentration, hydrolysis time, and temperature [30]. The current study is an attempt to optimize the process variables to obtain the highest collagen yield per gram of fish scales. Various factor like acetic acid concentration (M), volume of acetic acid (mL), time (h), and Tris-Glycine buffer (mL) were optimized to achieve maximum yield of collagen. The results obtained were validated using analysis of variance (ANOVA) focusing on dominant factors Interaction effect between the variables were studied using regression model.

## 2. Results and Discussion

### 2.1. Assessing the Effective of Optimized Parameters on Collagen Yield

The effect of acetic acid concentration, volume of acid, time of soaking, and buffer on the extraction of collagen was studied by one variable at a time using Taguchi method.

#### 2.1.1. Effect of Acetic Acid Concentration on Collagen Extraction

**Figure 1:**
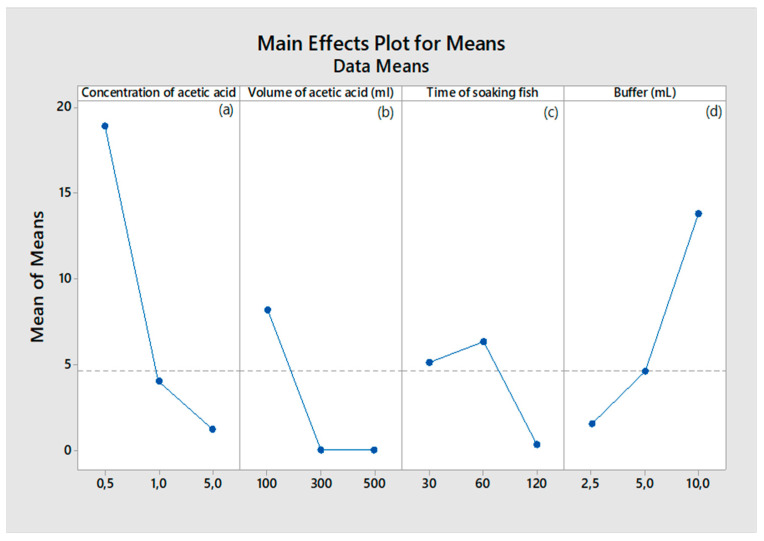
Effect of different parameters on collagen yield such as (**a**) acetic acid (molarity), (**b**) volume of acid (mL), (**c**) time of soaking (min) (**d**) buffer of Tris-Glycine (mL).

The effect of the acetic acid concentration (0.5–5.0 M) on collagen extraction was determined while keeping the other three variables constant. Figure 1a shows a steep decline in the mean response as the concentration of acetic acid increases from 0.5 M to 5 M. This indicates that higher acetic acid concentrations significantly reduce collagen yield. This might be due to the excessive acid degrading the collagen or affecting its solubility. This agrees with prior research showing that high concentrations of acid can degrade collagen, affecting the yield during the extraction process [4].

#### 2.1.2. Effect of Volume of Acetic Acid on Collagen Extraction

The effect of the volume of acetic acid (100–500 mL) on collagen extraction was determined while keeping the other three variables constant. Figure 1b shows that there is a decline in the mean response as the volume of acetic acid increases from 100 mL to 500 mL, with minimal changes between 300 mL and 500 mL. This implies that larger volumes of acetic acid may not necessarily improve the demineralization or collagen extraction, potentially due to saturation effects. Kim and Mendis [31] mention that excessive volumes of acids can dilute the reagents and lower efficiency, without increasing the extraction yield proportionately.

#### 2.1.3. Effect of Time of Soaking on Collagen Extraction

The effect of the volume of acetic acid (60–120 min) on collagen extraction was determined while keeping the other three variables constant. Figure 1c shows an initial increase in the mean response at 60 min of soaking, followed by a drop at 120 min, implying that soaking fish scales for 60 min appears optimal, while prolonged soaking (120 min) could lead to collagen degradation.

#### 2.1.4. Effect of Buffer Concentration on Collagen Extraction

The effect of the buffer (2.5–10 mL) on collagen extraction was determined while keeping the other three variables constant. Figure 1d shows an increase in the mean response with increasing buffer volume, peaking at 10 mL. The finding suggests that a higher volume of Tris-Glycine buffer improves collagen yield, likely by stabilizing the pH and preventing collagen degradation during extraction. Tris-Glycine buffers are known for maintaining pH stability during protein extractions [32], which can enhance collagen yield. Presumably, buffer solutions help stabilize the pH during extraction, ensuring that collagen remains intact, which agrees with research indicating that buffers are crucial for maintaining collagen structure during processing [33,34].

### 2.2. Experimental Factors Affecting the Extraction of Collagen

Table 1 shows the results of a Taguchi method experiment with four factors affecting the extraction of collagen. The Delta values represent the difference between the highest and lowest responses for each factor, and the Rank indicates the order of importance of the factors based on their impact on collagen yield. The concentration of acid has the largest effect on collagen extraction, as indicated by the highest delta value (17.7105). The response decreases sharply with increasing acid concentration. Therefore, lower concentrations of acid (Level 1) produce the highest collagen yield, and increasing the concentration results in diminished extraction efficiency. This aligns with the literature showing that higher concentrations beyond 0.6 M can degrade collagen, reducing yield [4,35].

The volume of acetic acid has a moderate effect on the collagen yield. The highest yield is observed at Level 1 (100 mL), but increasing the volume beyond this point results in no significant improvement (Levels 2 and 3 show no response). This could be due to the saturation of the solution, where increasing the volume of acetic acid beyond a certain threshold provides no additional benefit to the extraction process. Soaking time has a relatively smaller impact on collagen yield compared to the acid concentration and buffer volume. The optimal time for soaking is 60 min (Level 2), with a slight improvement over shorter times (Level 1: 30 min). However, soaking for longer periods (Level 3: 120 min) results in a sharp decline in yield, potentially due to collagen degradation after extended exposure to acidic conditions [36].

The buffer volume has the second-largest impact on collagen extraction, with the highest yield observed at Level 3 (10 mL). This shows that larger buffer volumes (10 mL) significantly enhance collagen yield, likely by stabilizing the pH during the extraction process, protecting the collagen from degradation. This finding is consistent with the literature on buffer use in biochemical extractions, where adequate buffer volume ensures pH stability and prevents enzymatic degradation [31,34].

### 2.3. ANOVA Model for Collagen Extraction Optimization

The ANOVA value in Table 2 provides insight into the significance of each factor affecting collagen extraction using the Taguchi method. The regression model is statistically significant with a *p*-value of 0.000, indicating that the factors included in the model (concentration of acetic acid, volume of acetic acid, time of soaking, and buffer) explain a significant amount of the variation in collagen yield. The high F-value (21.87) also indicates a strong overall effect of the factors on the outcome. The finding suggests that the concentration of acetic acid and buffer volume are the most significant factors influencing collagen yield, as evidenced by their high F-values and very low *p*-values (0.001 for both). Controlling these two factors is critical for optimizing collagen extraction. On the other hand, the volume of acetic acid has a moderate effect (*p* = 0.039), while soaking time does not significantly impact collagen yield within the range tested (*p* > 0.05). This result contradicts other previous studies, which show that time, concentration of the acid, and temperature are significant parameters in the collagen yield [26,37]. The plausible explanation may be connected to the differences between fish scales as used in the present study and fish skins reported in the literature.

### 2.4. Responses Modelling

Mathematical relationships between the responses and the studied factors are developed and presented by the following equations shown in Table 3. The model explains a large portion of the variance in the data (*R*^2^ = 73.84%), and all variables show acceptable levels of multicollinearity (VIF values). The concentration of acetic acid and buffer volume are the most significant factors affecting collagen yield, with concentration negatively affecting yield (−1.504) and buffer volume positively affecting it (1.152). The volume of acetic acid plays a moderate role in reducing collagen yield (−0.01330), while the time of soaking has an insignificant effect within the tested range (0.0127). These findings are consistent with previous studies, where optimizing acid concentration and maintaining buffer stability were crucial for maximizing collagen yield [38].

**Figure 2:**
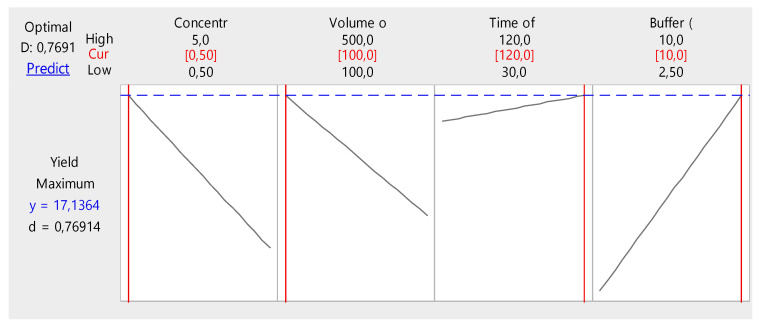
Optimized regression model.

Figure 2 further provides a graphical representation of an optimization process for the collagen yield experiment, where different factors (concentration of acetic acid, volume of acetic acid, time of soaking, and buffer volume) are being evaluated for their effects on the yield. The optimal factor combination for achieving the highest collagen yield is represented in the plot: concentration of acetic acid (0.50), volume of acetic acid (100 mL), time of soaking (around 120 min), and buffer volume: close to the high end (10 mL). This suggests that minimizing the concentration and volume of acetic acid while maximizing the buffer volume and keeping the soaking time relatively high will result in the highest collagen yield. The predicted maximum yield of 17.1364% reflects a high collagen yield for the given experimental setup. 

### 2.5. Amino Acid Quantification and Characterization

SDS-PAGE Characterization of Collagen

**Figure 3:**
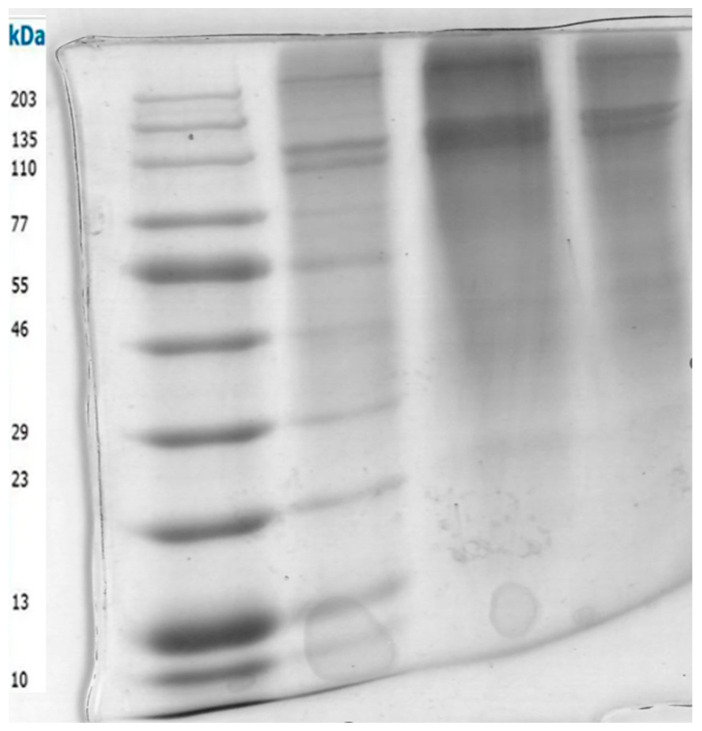
Lane 1—Protein Ladder, Lane 2–4—Collagen samples prepared in different buffers.

Figure 3 shows the SDS-PAGE of type I collagen extracted from marine samples. Lane 1: molecular weight marker (MW); Lanes 2–4: extracted type I collagen with different substrates (25 mM Tris-HCl buffer, pH 8.0, 2% SDS, 2% dithiothreitol, 20% glycerol, and 0.02% bromophenol blue). On SDS-PAGE, three bands are commonly found for collagen type I, which refers to the basic structure of monomeric alpha-1 and alpha-2 monomers, as well as the common motif of regular collagen secondary structures of the β-band and γ-band. The alpha bands of type I collagen are approximately 100–130 kDa, while the beta band is approximately 250 kDa [39]. The β-band represents a dimer representing two alpha chains crosslinking with each other, and the γ-band represents three alpha chains, which are visible in the upper part of the gel [40]. SDS-PAGE revealed the typical pattern for type I collagen, with bands at apparent molecular weights of 208 kDa (b), 130 kDa (a1), and 116 kDa (a2). We detected significant collagenase activity using (1) collagen dissolved in acetic acid and 5% SDS (Lane 1), (2) collagen boiled in sodium chloride at 80 °C (Lane 2), and (3) acetic acid only (Lane 3). Collagenases cleave type I collagen at specific sites in the a-chain, leading to the formation of 1/4 C-terminal and 3/4 N-terminal fragments [41]. Amongst the detected collagenase activity, collagen boiled in sodium chloride at 80 °C (Lane 2) gave the best activity; hence, it was further purified by 80% ammonium sulfate precipitation and run on 10% SDS-PAGE and provided the picture below. Collagen is the most abundant structural protein found in humans and mammals, particularly in the extracellular matrix (ECM) [39]. Its primary function is to hold the body together. The collagen superfamily of proteins includes over 20 types that have been identified [41]. Although collagen type I is the major component in many tissues. Type I collagen is commonly recognized as the gold standard biomaterial for the manufacturing of medical devices for healthcare-related applications. In recent years, with the final aim of developing scaffolds with optimal bioactivity [40].

**Figure 4:**
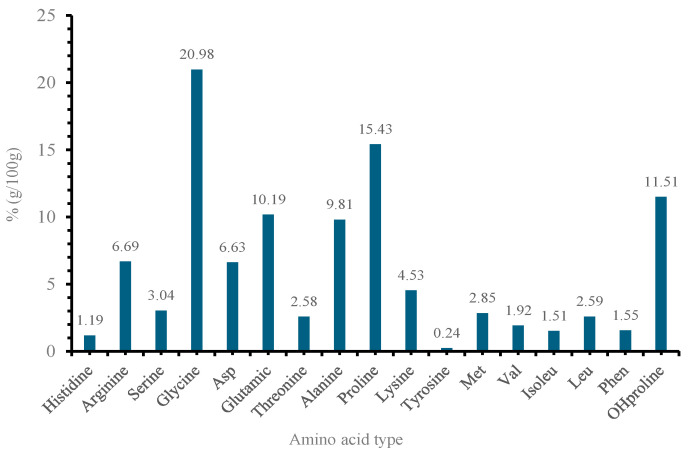
Type and concentration of amino acids in the extracted collagen.

Amino acids are the building blocks of proteins, and their distribution is crucial in determining the overall characteristics of the protein. The composition of the amino acids in 100 g revealed 17 different types of amino acids (Figure 4). The high percentages of glycine (20.98%), proline (15.43%), and hydroxyproline (11.51%) suggest a significant presence of structural proteins like collagen. According to Gauza–Włodarczyk, Kubisz [42], glycine determines the regular structure of the collagen chain and allows for the formation of stabilizing bonds between chains. In addition, the presence of hydroxyproline is also practically utilized for the quantitative assessment of collagen [42].

**Figure 5:**
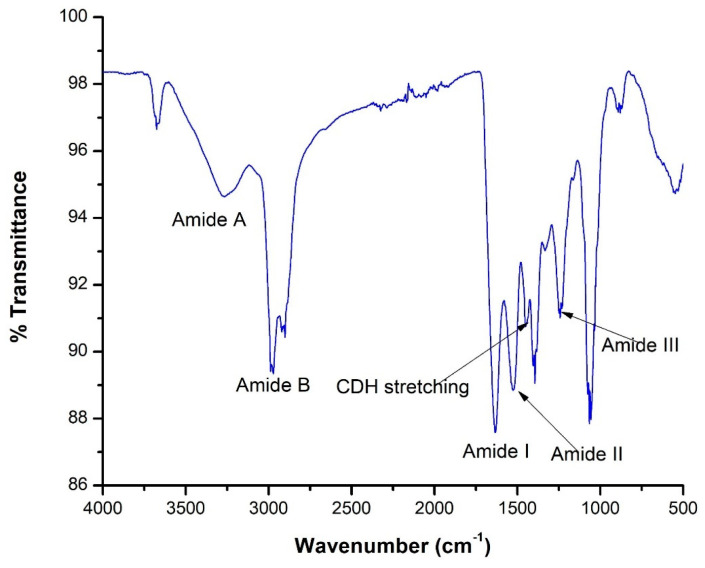
FTIR spectrum of extracted collagen.

The FTIR spectrum in Figure 5 displays several peaks corresponding to distinct amide bands (A, B, I, II, and III), which are characteristic of collagen due to their association with peptide bonds in protein structures [10,43]. For instance, the amide A band appears at approximately 3293 cm^−1^, representing N-H stretching vibrations typical of peptide bonds found in proteins like collagen. The amide B band, at approximately 2927 cm^−1^, is linked to asymmetrical N–H stretching vibrations, also related to peptide linkages. The amide I band, observed at approximately 1611 cm^−1^, is crucial for protein analysis, as it mainly corresponds to C=O stretching vibrations in peptide bonds and provides insights into the protein’s secondary structure. The amide II band, at approximately 1523 cm^−1^, is associated with N–H bending and C–N stretching vibrations, while the amide III band, approximately 1300 cm^−1^, involves complex vibrations of C–N stretching and N–H bending, offering additional details about the protein’s conformation. Additionally, C–H deformation peaks of approximately 1451 cm^−1^ are linked to C–H stretching vibrations, which originate from various side chains in the collagen molecule [10].

## 3. Materials and Methods

Roughly 100 kg of fresh fish scales were obtained from a local fish shop in Durban, South Africa. The samples were brought to the laboratory and rinsed with distilled water and 5 mL of household bleach. Sigma-Aldrich (Johannesburg, South Africa), a chemical manufacturer business, provided food-grade acetic acid. All of the reagents and chemicals used were of the highest grade.

### 3.1. Preparation and Extraction of Fish Scale Collagen (FSC) from Fish Scales

Using the collected fish scales, collagen was extracted through acid hydrolysis. All preparation and chemical extraction of the fish scales was performed at the Biochemistry and X Laboratory (Durban University of Technology, Durban, and Mintek, Randberg, South Africa). Following defrosting to room temperature to facilitate bacteria removal, the scales were air-dried for 2 days to eliminate excess water. FSC was extracted according to a well-established protocol involving demineralization and acid hydrolysis [44]. The scales were treated with 0.1 N NaOH for 2 days to eliminate noncollagenous proteins and pigments. After washing and drying to a constant weight, collagen extraction was carried out using varied parameters. Collagen extraction involved soaking 100 g of scales in varying concentrations of acetic acids (0.5 M, 1 M, and 5 M) in volumes of acid (100, 300, and 500) at different soaking times (30 min, 60 min, and 120 min) at 4 °C with intermittent stirring. The solution underwent centrifugation at 3000 rpm for 30 min at 4 °C. Supernatants were salted out by adding 9% NaCl, stirred automatically for 1 day, and suspended in a Tris-Glycine buffer at different concentrations (2.4 mL, 5 mL, and 10 mL). Pellets were collected via centrifugation, washed with distilled water, and subjected to dialysis using a dialysis membrane (MWCO 12–14 kDa) to eliminate residual salts. The resulting material was freeze-dried at −80 °C under vacuum conditions. Characterization of the resulting collagen was conducted using different analytical techniques.

### 3.2. Optimization of the Extraction Parameters

As advocated in the literature, the Design of Experiments (DOE) was used to determine the number of screening experiments to optimize the parameters [45]. A four-factor-three-level at the center point and 36 experiments. The optimal experimental conditions are determined by comparing the mean averages. Fisher RA emphasized the importance of identifying the optimal combination of factors and levels in Design of Experiments (DOE) to achieve desired outcomes. The results are validated through Analysis of Variance (ANOVA) focusing on dominant factors, as indicated by prior studies [46,47]. Table 4 illustrates the primary factors and their corresponding levels, while Table 5 outlines the extraction conditions. Utilizing MINITAB 17 software, this methodology enables the evaluation of test outcomes in the form of signal-to-noise ratios (SN).

Collagen yields were calculated as follows:

(1) $$Yield\;\left(\%\right)=\;\left(\;\frac{Dry\;collagen\;weight}{Dry\;scale\;weight}\right)\times 100$$

where: dry collagen weight = the weight of collagen obtained after extraction and drying; dry scale weight = the original weight of the dry fish scales used in the extraction process. Yield (%) refers to the efficiency of collagen extraction from fish scales. It is expressed as a percentage of the collagen obtained relative to the original amount of fish scales used.

### 3.3. SDS-PAGE Analysis

SDS-PAGE was carried out as per the Laemmli 1970 method [48] to estimate the molecular weight of the enzyme and visualize and evaluate protein purification efficiency with the optimized collagen yield. Before SDS-PAGE analysis, the samples (40 µg in 15 µL distilled water) were prepared by mixing them with 5 µL of sample buffer (25 mM Tris-HCl buffer, pH 8.0, 2% SDS, 2% dithiothreitol, 20% glycerol, and 0.02% bromophenol blue). The samples were then heated at 100 °C for 5 min and cooled at room temperature. Samples were loaded on the gel (5% stacking gel and 10% resolving gel) and electrophoresed at 20 mA per gel at room temperature until the dye front reached the end of the gel. Coomassie Brilliant Blue R250 (0.25% dye, 10% acetic acid, 45% ethanol in distilled water) was used to stain the gels. The gels were destained (7.5% acetic acid, 5% ethanol in distilled water) and photographed with a Gel Doc XR system (BioRad, Thermo Fisher Scientific Inc., Waltham, MA, USA).

### 3.4. Protein Estimation

The Kjeldahl method was used to determine the amino acid content of the extracted optimized collagen, while the Stegmann method [49] was applied to measure the hydroxyproline concentration. An automatic amino acid analyzer was employed to obtain the results for the amino acid composition of collagen, as presented in Figure 2. Since amino acids serve as the fundamental components of proteins, their distribution plays a key role in defining the protein’s overall properties. The determination of amino acid content had a relative error of 5%, implying low variability.

### 3.5. Fourier Transform Infrared Assessment

The Perkin Elmer Universal ATR was used to analyze the structural changes in the PU composite. A background scan was conducted before the actual measurements. Each prepared sample was then placed in a sample holder and scanned with a resolution of 4 cm^−1^ over the wavenumber range of 400–4500 cm^−1^.

## 4. Conclusions

In this study, the Taguchi methodology was implemented, and the optimal conditions (concentration of acetic acids, buffer, volume, and time) to obtain the highest collagen yield (per 100 g of fish scales) were determined. The finding suggests that the maximum collagen yield of 17.14 ± 0.05 mg/g of fish scales was achieved under the optimal conditions. This study has shown that an improved yield of collagen was obtained when the concentration of acetic acid was 0.5 M, the volume of acid was 100 mL, the soaking time was 120 min, and the buffer concentration was 10 mL. It was found that three of the variables, except time of soaking, showed a significant effect on the extraction of collagen from the fish scales, and a positive relation was observed between acetic acid concentration and Tris-Glycine Buffer variables. The obtained mathematical model had an *R*^2^ value of 73.84% and a *p*-value of <0.0001, which implicated a moderate agreement of the experimental values of the yield of collagen from the fish scales and affirmed a good generalization of the mathematical model. FTIR analysis confirmed the presence of characteristic collagen peaks at 1611 cm^−1^ (amide I), 1523 cm^−1^ (amide II), and 1300 cm^−1^ (amide III), indicating the successful extraction of type I collagen. SDS-PAGE analysis revealed a protein banding pattern consistent with the molecular weight of collagen.

Although this study successfully determined the optimal conditions for collagen extraction using the Taguchi methodology and achieved a maximum collagen yield of 17.14 ± 0.05 mg/g of fish scales, it did not perform an experimental validation of the optimized process. Experimental validation involves conducting trials under the identified optimal conditions to confirm whether the theoretical results align with experimental outcomes. Future studies would prioritize experimental validation to ensure the reliability and reproducibility of the optimized process for practical applications. This step would provide a comprehensive understanding of how the optimized variables affect collagen yield in real-world scenarios.

## Figures and Tables

**Table 1:** Response for means of collagen yield.

Level	Concentration of Acid	Volume of Acidic Acid (mL)	Time of Soaking (Minutes)	Buffer (mL)
1	18.9100	8.1615	5.1312	1.5647
2	4.0194	0.0000	6.3310	4.5850
3	1.1995	0.0000	0.3480	13.7750
Delta	17.7105	8.1615	5.9830	12.2103
Rank	1	3	4	2

**Table 2:** ANOVA model for collagen yield extraction.

Source	Df	Adj SS	Adj MS	*F*-Value	*p*-Value
Regression	4	1750.99	437.747	21.87	0.000
Concentration of acetic acid	1	250.47	250.471	12.52	0.001
Volume of acetic acid (mL)	1	92.54	92.545	4.62	0.039
Time of soaking (min)	1	4.78	4.778	0.24	0.629
Buffer (mL)	1	243.41	243.410	12.16	0.001
Error	31	620.42	20.014		
Lack-of-fit	18	429.93	23.885	1.63	0.187
Pure Error	13	190.49	14.653		
Total	35	2371.41			

Df = degree of freedom, Adj SS = Adjusted Sum of Squares, Adj MS = Adjusted Mean Squares, *p*-Value = Probability value, *F*-Value = Analysis of Variance (ANOVA) value.

**Table 3:** The mathematical models of the responses.

Response	The Obtain Model	Factors	Coef	SE Coef	*R* ^2^	*T*-Value	*p*-Value	VIF
Yield (%)	6.17 − 1.504 Concentration of acetic acid − 0.01330 Volume of acetic acid (mL) + 0.0127 Time of soaking fish + 1.152 Buffer (mL)	Constant	6.17	3.88	73.84	1.59	0.122	
		Concentration of acetic acids	−1.504	0.425		−3.54	0.001	1.41
		Volume of acetic acid	−0.01330	0.00619		−2.15	0.039	1.61
		Time of soaking	0.0127	0.0260		0.49	0.629	1.18
		Buffer	1.152	0.330		3.49	0.001	2.22

*R*^2^ = coefficient of determination, VIF = Variance Inflation Factor, Standard Error of the Coefficient.

**Table 4:** Coded values and independent variables used for optimization.

Factors	Symbols	Coded Level		
		−1	0	+1
Concentration of acetic acid	A	0.5	1	5
Volume of acetic acid (mL)	B	100	300	500
Time of soaking fish	C	30	60	120
Buffer (mL)	D	2.5	5	10

**Table 5:** Plan of experiments (extraction conditions) as per 36 runs.

Run	Concentration of Acetic Acid	Volume of Acetic Acid (mL)	Time of Soaking Fish	Buffer (mL)	Yield
1	0.5	100	30	2.5	0
2	0.5	100	60	2.5	0
3	0.5	100	120	2.5	0
4	0.5	300	30	2.5	0
5	0.5	300	60	2.5	0
6	0.5	300	120	2.5	0
7	0.5	500	30	2.5	0
8	0.5	500	60	2.5	0
9	0.5	500	120	2.5	0
10	5	100	30	2.5	0
11	5	100	60	2.5	21.73
12	5	100	120	2.5	3.48
13	5	300	30	2.5	0
14	1	300	60	2.5	0
15	1	300	120	2.5	0
16	1	500	30	2.5	0
17	5	500	60	2.5	0
18	5	500	120	2.5	0
19	0.5	100	30	5	0
20	0.5	100	30	5	0
21	0.5	100	60	5	4.53
22	5	100	60	5	0
23	5	100	60	5	11.94
24	5	100	60	5	11.04
25	0.5	100	30	10	11.64
26	0.5	100	60	10	11.85
27	0.5	100	60	10	10.89
28	5	100	30	10	11.56
29	5	100	30	10	14.81
30	5	100	60	10	11.5
31	0.5	100	30	10	9.19
32	0.5	100	30	10	20.9
33	0.5	100	60	10	22.28
34	5	100	30	10	20.13
35	5	100	30	10	22.03
36	5	100	60	10	20.28

## Data Availability

Data can be made available by the author upon request.
